# Supplementary material for: Identification of photoperiod-induced specific miRNAs in the adrenal glands of Sunite sheep (Ovis aries)
Source: Front Vet Sci. 2022 Jul 22;9:888207. doi: 10.3389/fvets.2022.888207 (PMC9354845; doi:10.3389/fvets.2022.888207)
Supplement: Supplementary file 1 [file Data_Sheet_1.ZIP › Supplementary file/miRDeep2 file.zip!/miRDeep2 file/Novel_Structure/SPLP42A1/Novel_187.pdf]

Provisional ID	:	NC_019475.2_24331
Score total	:	97.5
Score for star read(s)	:	3.9
Score for read counts	:	89.9
Score for mfe	:	2.2
Score for randfold	:	1.6
Score for cons. seed	:	
Total read count	:	188
Mature read count	:	184
Loop read count	:	0
Star read count	:	4

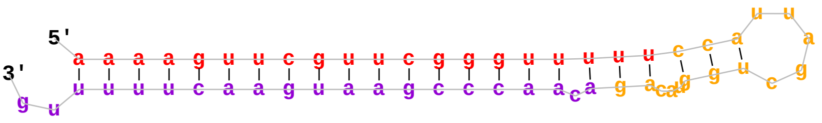

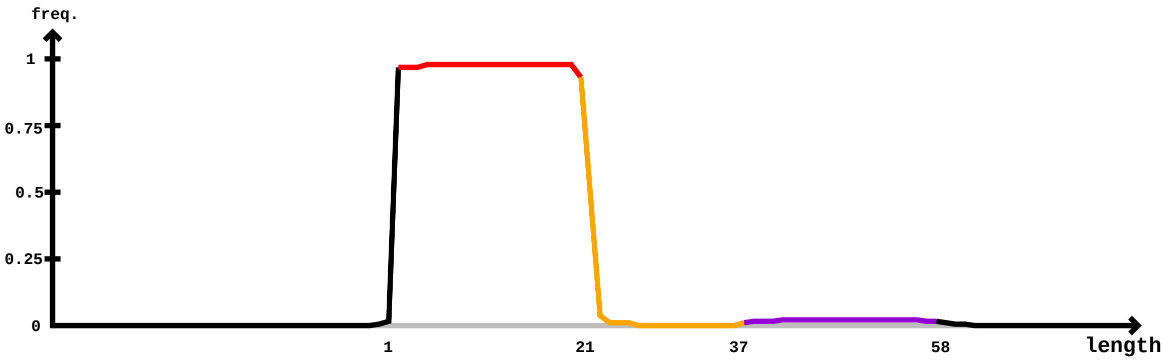

Mature **Star**

```
5'- cccugggggaggaagggauguacuggguuggcccaaaaguucguucggguuuuuccauuagcugguacagacaacccgaaugaacuuuuuggccaauucaauauuuccagc   -3'   obs
    cccugggggaggaagggauguacuggguuggcccaaaaguucguucggguuuuuccauuagcugguacagacaacccgaaugaacuuuuuggccaauucaauauuuccagc         exp
    .(((.....)))...(((.(((.((((((((((.(((((((((((((((((((((((.....)))...))).))))))))))))))))).)))))))))).))).)))...     reads   mm    sample
    ................................cAaaaaguucguucggguuuuu.........................................................     1       1     seq
    .................................Aaaaaguucguucggguuuuuc.......................................................     2       1     seq
    ..................................aaaaguucguucggguuC...........................................................     2       1     seq
    ..................................aaaaguucguucggguuu...........................................................     7       0     seq
    ..................................aaaaguucguucggguuuuu.........................................................     99      0     seq
    ..................................aaaaguucguucggguuuuuc........................................................     66      0     seq
    ..................................aaaaguucguucggguuuuucU.......................................................     5       1     seq
    .....................................aguucAuucggguuuuuccauu....................................................     2       1     seq
    ......................................................................acaacccAaaugaacuuuu......................     1       1     seq
    ......................................................................acaacccgaaugaacuuuuug....................     1       0     seq
    .......................................................................caacccgaaugaacuuuuug....................     1       0     seq
    ..........................................................................cccgaaugaacuuuuuggcA.................     1       1     seq
```
